# Proteogenomic analysis reveals exosomes are more oncogenic than ectosomes

**DOI:** 10.18632/oncotarget.3801

**Published:** 2015-04-12

**Authors:** Shivakumar Keerthikumar, Lahiru Gangoda, Michael Liem, Pamali Fonseka, Ishara Atukorala, Cemil Ozcitti, Adam Mechler, Christopher G. Adda, Ching-Seng Ang, Suresh Mathivanan

**Affiliations:** ^1^ Department of Biochemistry, La Trobe Institute for Molecular Science, La Trobe University, Melbourne, Victoria, Australia; ^2^ Department of Chemistry, La Trobe Institute for Molecular Science, La Trobe University, Melbourne, Victoria, Australia; ^3^ Bio21 Institute, University of Melbourne, Victoria, Australia

**Keywords:** exosomes, ectosomes, proteogenomics, extracellular vesicles, integrated OMICs analysis

## Abstract

Extracellular vesicles (EVs) include the exosomes (30-100 nm) that are produced through the endocytic pathway via the multivesicular bodies and the ectosomes (100-1000 nm) that are released through the budding of the plasma membrane. Despite the differences in the mode of biogenesis and size, reliable markers that can distinguish between exosomes and ectosomes are non-existent. Moreover, the precise functional differences between exosomes and ectosomes remains poorly characterised. Here, using label-free quantitative proteomics, we highlight proteins that could be exploited as markers to discriminate between exosomes and ectosomes. For the first time, a global proteogenomics analysis unveiled the secretion of mutant proteins that are implicated in cancer progression through tumor-derived EVs. Follow up integrated bioinformatics analysis highlighted the enrichment of oncogenic cargo in exosomes and ectosomes. Interestingly, exosomes induced significant cell proliferation and migration in recipient cells compared to ectosomes confirming the oncogenic nature of exosomes. These findings ascertain that cancer cells facilitate oncogenesis by the secretion of mutant and oncoproteins into the tumor microenvironment via exosomes and ectosomes. The integrative proteogenomics approach utilized in this study has the potential to identify disease biomarker candidates which can be later assayed in liquid biopsies obtained from cancer patients.

## INTRODUCTION

Intercellular communication is a highly conserved process by which cells receive and transmit signals [1]. It was generally thought that intercellular communication is mediated by direct cell-to-cell contact, plasma membrane and soluble secreted proteins. In recent years, a new mode of intercellular communication mediated by extracellular vesicles (EVs) has gained prominence [2, 3]. These membranous EVs secreted by the host cells activate target cells close to the host cells' proximity and more importantly mediate long range signaling events [4-6]. Based on the mode of biogenesis, EVs can be broadly classified as exosomes, ectosomes and apoptotic bodies (from apoptosis) [7]. Exosomes are small membranous vesicles of 30–100 nm in diameter that are secreted by a variety of cells when the multivesicular bodies (MVBs) fuse with the plasma membrane [8]. On the contrary, ectosomes or shedding microvesicles are vesicles of larger size (100-1000 nm in diameter) that buds off directly from the plasma membrane [9, 10].

It is generally accepted that currently available EV purification methods seldom allow for complete separation of exosomes and ectosomes [8, 11, 12]. Hence, multiple studies have analysed the EV subtypes together and have not worked with a pure homogeneous population [13]. As a result of the challenges in the isolation of EV subtypes to homogeneity, molecular profiling and functional characterization studies pertaining to EV subtypes are limited. Among the EV subtypes, exosomes have been characterized by multiple groups [7] while ectosomes remains understudied. Barring the information on the purported mode of biogenesis and size, very little is known about the buoyant density and the protein composition of ectosomes. The lack of reliable protein markers that can discriminate between exosomes and ectosomes impedes the field of EVs as the specific function could not be attributed to the exact population of EVs. Furthermore, deficiency in the number of studies that are aimed to characterize ectosomes has also resulted in limited knowledge on the biogenesis and the functional insights of ectosomes. Hence, investigations on the molecular cargo of ectosomes and exosomes may provide valuable information on the biogenesis, cargo sorting and functional roles.

Here, we isolated exosomes and ectosomes from neuroblastoma cells by ultracentrifugation and OptiPrep^TM^ density gradient centrifugation. A follow up label-free quantitative proteomics analysis highlighted distinct markers of exosomes and ectosomes. For the first time, a global integrated proteogenomics analysis of exosomes and ectosomes revealed mutant/aberrant proteins that are secreted via EVs. Using an integrated computational and experimental approach, we uncovered the oncogenic potential of exosomes and ectosomes secreted by cancer cells.

## RESULTS

### Isolation and characterisation of exosomes and ectosomes

**Figure 1:**
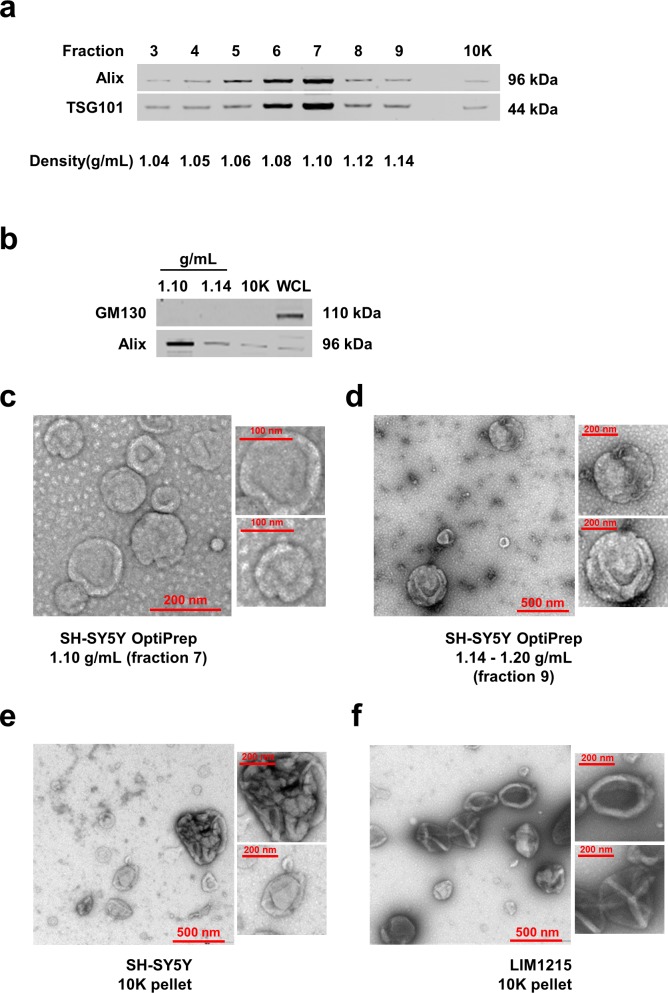
Isolation and characterization of EVs (**a**) Western blot analysis (10 μg) of fractions of increasing density obtained by OptiPrep^TM^ density gradient centrifugation (from SH-SY5Y cells) showed the presence of Alix and TSG101 in fractions 3-9 with a clear enrichment in fraction 7 (1.10 g/mL). Pellet obtained from 10,000 *g* also contained detectable amount of Alix and TSG101. (**b**) Western blot of GM130 (Golgi marker) and Alix in EVs isolated from 1.10 and 1.14 g/mL densities, 10,000 g pellet and WCL is shown. The absence of GM130 in fractions 7, 9 and 10K confirms the depletion of contaminating vesicles arising from apoptosis. (**c**) TEM images of EVs isolated from SH-SY5Y cells by OptiPrep^TM^ gradient corresponding to the density 1.10 g/mL showed vesicles in the range of 30-100 nm diameter consistent with exosomes. (**d**) TEM images of EVs isolated from SH-SY5Y cells by OptiPrep^TM^ gradient corresponding to the density 1.14-1.20 g/mL showed vesicles more than 200 nm in diameter. (**e**) TEM images of vesicles recovered from 10,000 *g* pellet (10K) showed aggregates and large EVs secreted by SH-SY5Y neuroblastoma and (**f**) LIM1215 colorectal cancer cells.

To isolate EVs simultaneously, SH-SY5Y neuroblastoma cells were cultured for 24 h. As apoptotic bodies may confound the interpretation of the data, Trypan blue assay and Western blot analysis were performed on SH-SY5Y cells to monitor the cell death. As shown in Supplementary Fig. 1, cell viability was more than 91% and no cleavage of PARP-1 or caspase 3 were observed in SH-SY5Y cells at the time of the collection of the conditioned media [14]. In order to isolate EVs, the conditioned media was subjected to differential centrifugations followed by ultracentrifugation, and separated by discontinuous iodixanol density gradient (OptiPrep™) centrifugation. Fractions of increasing densities were subjected to Western blot analysis using Alix and TSG101 antibodies to identify exosome enriched samples [3, 15, 16]. Alix and TSG101, components of the endosomal sorting complex required for transport (ESCRT) machinery, are often secreted via exosomes by a variety of cells and hence considered as exosomal markers [3]. As shown in Fig. 1a, the exosomal markers were enriched in fractions 6 and 7 corresponding to the density of 1.08-1.10 g/mL. Fractions 1-2 and 10-12 were not subjected to Western blotting due to low protein yield (<10 μg). Though equal amounts of protein (10 μg) were analysed by Western blotting, Alix and TSG101 were not enriched in fraction 9 (1.14 g/mL). In addition to the fractions separated by OptiPrep™ density gradient centrifugation, the pellet obtained directly after the 10,000 *g* centrifugation (10K) was also subjected to Western blotting. As shown in Fig. 1a, the 10K pellet also contained low but detectable amounts of Alix and TSG101.

The density for exosome enriched fraction was consistently 1.10 g/mL irrespective of multiple biological replicates. However, the higher density fraction that contained more than 20 μg of protein was ranging from 1.14-1.20 g/mL when the isolation procedures were repeated. As fraction 7 (1.10 g/mL) was the most enriched for exosomal markers, the sample was used for further analysis. As we intended to characterize larger vesicles, higher density fraction 9 (1.14-1.20 g/mL) was utilized for subsequent analysis. To reconfirm the absence of contaminants due to cell death, Western blotting was performed for GM130, a Golgi apparatus marker that is considered to be absent in EVs [12]. As shown in Fig. 1b, GM130 could not be detected in either fraction 7, 9 or 10K pellet confirming the absence of apoptotic cell debris. As 10,000 *g* centrifugation will mostly pellet larger vesicles such as ectosomes, the presence of the so-called exosomal markers Alix and TSG101 in 10K pellet emphasizes the need to identify unique markers to distinguish between exosomes and ectosomes.

### Microscopic analysis further confirmed the presence of EVs with different morphological properties

**Figure 2:**
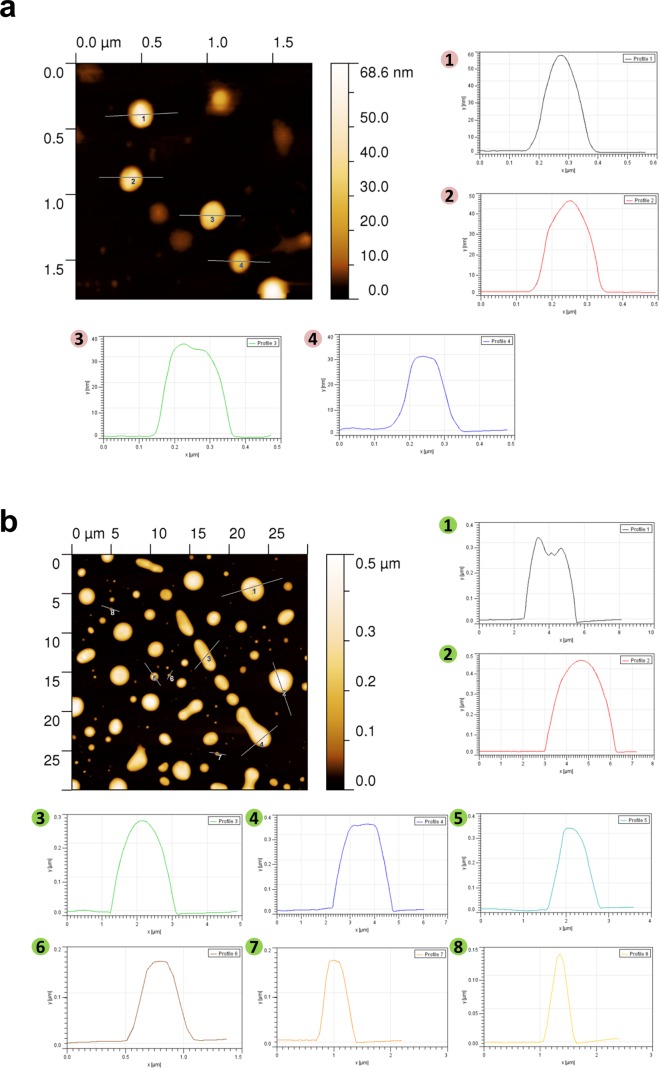
AFM imaging based characterization of EVs isolated by OptiPrep™ density gradient centrifugation (**a**) AFM images of fraction 7 (1.10 g/mL) isolated by OptiPrep^TM^ density gradient centrifugation showed vesicles in the range of 30-70 nm. Four profile images (1-4) of the vesicle diameter are also depicted. (**b**) AFM images of EVs obtained by OptiPrep^TM^ density gradient centrifugation corresponding to 1.14-1.20 g/mL showed enrichment of larger vesicles. Eight profile (1-8) images of the vesicle diameter are also depicted.

**Figure 3:**
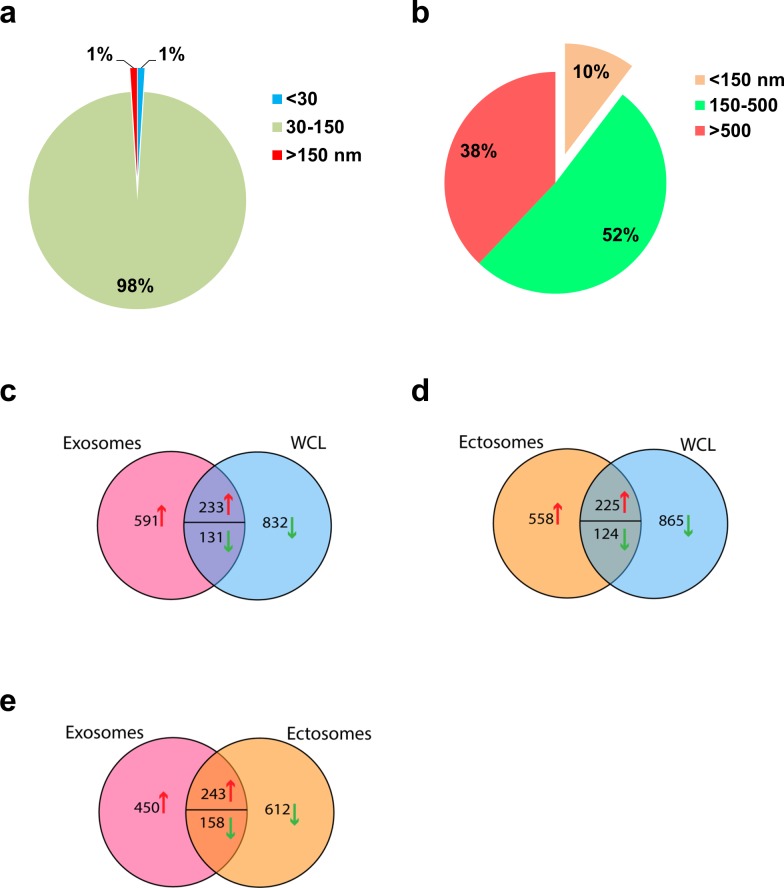
Vesicle size distribution and Venn diagram of total and differentially abundant proteins in exosomes, ectosomes and WCL (**a**) Pie chart representing the size distribution of vesicles in fraction 7 (1.10 g/mL). (**b**) Pie chart representing the size distribution of vesicles in fraction 9 (1.14-1.20 g/mL). (**c**) Label-free quantitative mass spectrometry-based proteomics analysis was performed on WCL, exosomes (1.10 g/mL) and ectosomes (1.14-1.20 g/mL). Venn diagram of differentially expressed proteins in exosomes and (**d**) ectosomes in comparison to WCL is displayed. (**e**) Venn diagram of differentially abundant proteins identified in exosomes and ectosomes showed 693 proteins enriched more than 2-fold in exosomes compared to ectosomes. On the contrast, 770 proteins were enriched more than 2-fold ectosomes compared to exosomes.

In order to further confirm the presence of exosomes (small EVs) and ectosomes (large EVs) by biophysical methods, fraction 7 (1.10 g/mL), fraction 9 (1.14-1.20 g/mL) and 10K pellet were subjected to transmission electron microscopy (TEM) and atomic force microscopy (AFM) analysis. A homogenous population of membranous vesicles within the range of 30–100 nm in diameter, characteristic of exosomes, was detected in fraction 7 (Fig. 1c). On the contrary, larger vesicles were enriched in fraction 9 (Fig. 1d) and 10K pellet (Fig. 1e). The observation of larger vesicles was also consistent in 10K pellet obtained from LIM1215 colorectal cancer cells (Fig. 1f). However, the 10K pellet had more proteinaceous background and the vesicles were much larger than fraction 9. From this result, it can be concluded that some of the larger vesicles (found in 10K pellet) could have ruptured during the high speed (100,000 *g*) ultracentrifugation and were not intact after density gradient centrifugation. In agreement with TEM, AFM imaging showed smaller vesicles in fraction 7 (1.10 g/mL) and larger vesicles in the higher density fractions (1.14-1.20 g/mL) (Fig. 2). While the vesicles appear to be partially flattened, it is estimated that majority of the vesicles are 30-70 nm in fraction 7 consistent with the size of exosomes [17] (Fig. 2a) and more than 200 nm in fraction 9 similar to ectosomes [9] (Fig. 2b). Size-based quantitation of the vesicles by AFM showed that 98% of the vesicles in fraction 7 were between 30-150 nm in diameter (Fig. 3a). On the contrary, 52% of the vesicles were between 150-500 nm in fraction 9 (Fig. 3b). Reliable AFM images for 10K pellet could not be obtained due to high proteinaceous background (data not shown). Overall, these results clearly suggest that the small and large EVs were enriched in fraction 7 and 9, respectively. Henceforth, fraction 7 and 9 corresponding to 1.10 g/mL and 1.14-1.20 g/mL are referred to as exosomes and ectosomes, respectively. However, it has to be noted that some smaller vesicles (<150 nm) were also observed in fraction 9 (1.14-1.20 g/mL) and 10K by the microscopic analysis. This could possibly be attributed either to the pelleting of a minor population of exosomes (trapped under larger vesicles during pelleting) or highly dense small vesicles that pellet at lower speeds or breakage of larger vesicles due to centrifugation.

### Proteomic analysis of SH-SY5Y cell-derived exosomes and ectosomes

In order to identify the proteins present in exosomes and ectosomes, LC-MS/MS-based label-free quantitative proteomics analysis was performed on the samples. Apart from exosomes and ectosomes, the whole cell lysate (WCL) and 10K pellet from SH-SY5Y cells were also subjected to proteomics analysis. Equal amounts of protein (20 μg) from the samples were separated by SDS-PAGE, gel bands were excised, reduced, alkylated and digested with trypsin. The extracted tryptic peptides were analysed by LTQ-Orbitrap Velos mass spectrometer. The resulting MS/MS spectrum was searched using X!Tandem against human RefSeq protein database and the protein list (at a false discovery rate of <0.5% at the peptide level) was consolidated (Supplementary Table 1, data submitted in Vesiclepedia [7]). Compared to WCL, 824 proteins were of high abundance in exosomes samples (Fig. 3c). Similarly, 783 proteins were of high abundance in ectosomes compared to WCL (Fig. 3d). The identification of cellular low abundant proteins in exosomes/ectosomes suggests that protein cargo sorting into EVs could be a highly selective process. Interestingly, a total of 693 and 770 proteins were of high and low abundance in exosomes compared to ectosomes (Fig. 3e). Among these, more than 1,000 proteins were exclusively identified in either exosomes or ectosomes clearly suggesting that the two EV types have distinct proteomic profiles.

### Proteins implicated in exosome biogenesis and trafficking were depleted in ectosomes

**Figure 4:**
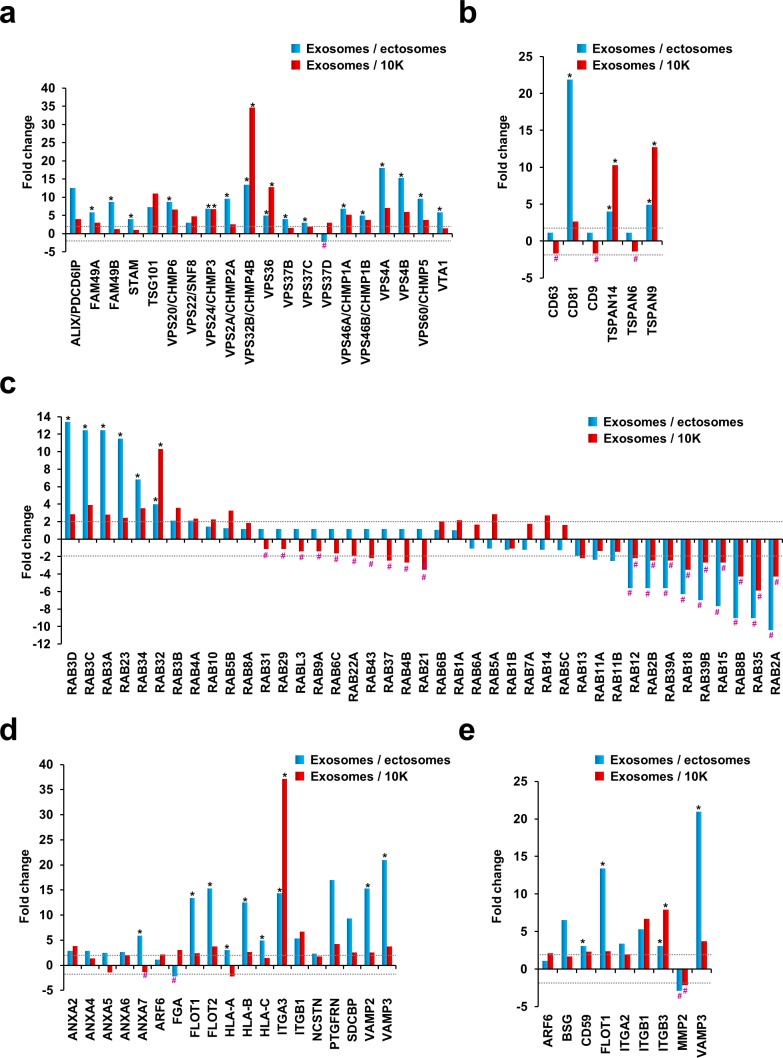
Fold change of proteins known to be involved in EV biogenesis, trafficking and membrane fusion Asterisk (*) represents proteins that are not detected in ectosomes and/or 10K while # represents proteins that are not detected in exosomes. Dotted line represents 2-fold cut off. (**a**) Histogram of proteins involved in ESCRT machinery showed that proteins involved in ESCRT were more than 2-fold abundant (except VPS37D) in exosomes compared to ectosomes. (**b**) Histogram of tetraspanins shows that exosomes are enriched with tetraspanins CD81, TSPAN14 and TSPAN9 compared to the ectosomes and 10K. CD63, CD9 and TSPAN6 were not enriched more than 2-fold in any data set (even though detected in exosomes with one peptide identification from multiple MS/MS spectra). (**c**) Among the RAB GTPases, subsets of them were uniquely identified in exosomes (*) while some others in ectosomes and/or 10K (#). The same set of RAB GTPases was uniquely present in both ectosomes and 10K while not detected in exosomes. (**d**) Proteins known to be involved in trafficking and membrane fusion were all enriched in exosomes except FGA and HLA-A. (**e**) A literature survey was carried out to identify proteins either involved in ectosome biogenesis or identified in ectosomes. The manually curated protein list was plotted as a histogram based on the proteomics data. Except MMP2, other proteins were not enriched in ectosomes or 10K.

It is well established that the ESCRT machinery is important for the sorting of ubiquitinated cargo and in the formation of exosomes [18]. Besides the regulation of exosomal biogenesis and cargo sorting, some of the ESCRT protein components are also detected in the secreted exosomes [3]. In order to assess the abundance of ESCRT protein machinery in exosomes, ectosomes and 10K pellet, histograms were plotted based on the fold change of the protein in exosomes compared to ectosomes or 10K. Consistent with Western blotting and previous studies [19], TSG101 and Alix were identified in exosomes isolated from neuroblastoma cells (Fig. 4a). Alix was 12.6-fold and 4-fold enriched in exosomes compared to ectosomes and 10K, respectively. Similarly, TSG101 was 7-fold and 11-fold enriched in exosomes compared to ectosomes and 10K, respectively. In accordance with Western blotting (Fig. 1b), the proteomic analysis also identified Alix and TSG101 in ectosomes and 10K. These observations further allude that Alix and TSG101 may not be reliable exosomal markers as previously thought [2, 20] and are rather exosome enriched as discussed in the Minimal Information for Studies on Extracellular Vesicles (MISEV) standards [12]. In accordance with the literature [2, 21], other ESCRT machinery proteins were enriched in exosomes compared to ectosomes and 10K (Fig. 4a). Among these, VPS24, VPS32 and VPS36 were exclusively identified in exosomes. In contrast, VPS37D was only detected in ectosomes. VPS37D is a component of ESCRT-1 complex and its role in the biogenesis of ectosomes is currently unknown. Collectively, these observations confirm that the ESCRT machinery plays a prominent role in exosome biogenesis and may have a minimal/or no role in regulating the biogenesis of ectosomes.

Tetraspanins, four transmembrane domain containing proteins [22], are heavily enriched in exosomes and are implicated in exosome biogenesis [3, 23]. In support of this, TSPAN9 and TSPAN14 were exclusively identified in exosomes (more than 4-fold enriched in exosomes compared to ectosomes and 10K) (Fig. 4b). Though detected in 10K pellet, CD81 was 22-fold and 2.7-fold enriched in exosomes compared to ectosomes and 10K. No marked difference in abundance could be observed for CD63, CD9 and TSPAN6 that were identified in the exosomal (<2 peptides) and 10K fractions by mass spectrometry. In addition to tetraspanins, Rabs, small GTPases which participate in vesicle docking and membrane fusion events, are also commonly detected in exosomes [3, 24]. Rabs form complexes with proteins involved in membrane trafficking through the endocytic system and are routinely used as markers of various endocytic compartments [25]. Interestingly, several members of the Rab GTPases were enriched in exosomes or ectosomes/10K (Fig. 4c). For instance, RAB12, RAB2B, RAB39A, RAB18, RAB39B, RAB15, RAB8B, RAB35 and RAB2A were enriched more than 2-fold in ectosomes and 10K compared to exosomes. Likewise, another set of RAB molecules including RAB3D, RAB3C, RAB3A, RAB23, RAB34, RAB32, RAB3B and RAB4A were more than 2-fold enriched in exosomes compared to both ectosomes and 10K. These observations suggest that two sets of RABs that may have unique roles in the biogenesis of distinct types of EVs could be present in the cell. Further experiments need to be performed to study the distinct functional roles of the RABs.

Proteomic analysis highlighted that exosomes are also enriched of lipid raft components such as flotillins. FLOT1 and FLOT2 were not detected in ectosomes and were 13- and 15-fold enriched in exosomes compared to ectosomes (Fig. 4d). Though flotillins were detected in 10K, exosomes were more than 2-fold enriched. Similarly, annexins A2, A4, A5, A6 and A7 were all enriched in exosomes compared to ectosomes. However, only annexin A2 was enriched in exosomes compared to 10K. Apart from these, syntenin, integrins and VAMP proteins were also highly enriched in exosomes compared to ectosomes and 10K. It has to be noted that, ITGA3, a protein critical in interaction with extracellular matrix, was exclusively identified in exosomes. These results collectively suggest that exosomes are enriched with proteins that are known to be involved in membrane transport and fusion.

**Figure 5:**
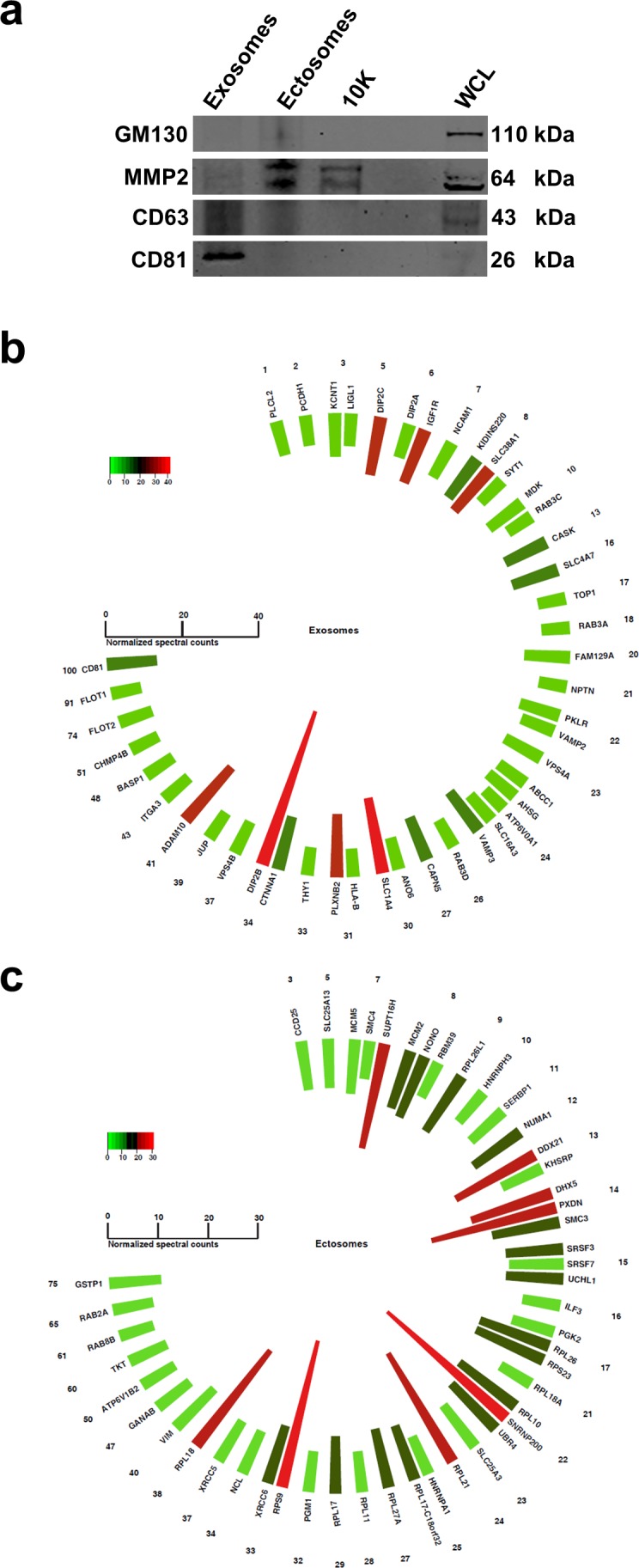
Western blotting analysis of exosomes and ectosomes and polar histogram of top 50 proteins abundant in exosomes and ectosomes (**a**) Western blot analysis of exosomes, ectosomes, 10K and WCL. CD81 is exclusively detected in exosomes while MMP2 is unique to ectosomes and 10K. CD63 was enriched in exosomes but was also detected in ectosomes. GM130 is absent in exosomes, ectosomes and 10K confirming the depletion of contaminating vesicles arising from apoptosis. (**b)** Normalised spectral counts of top 50 abundant proteins are displayed. The list is sorted by the number of times the particular protein is identified in Vesiclepedia. The numbers (outer circle) correspond to the number of studies reported in Vesiclepedia. The color scale represents the protein abundance level in terms of normalised spectral count. Top 50 abundant proteins in exosomes include CD81, flotillins and ADAM10 which are some of the proteins identified more often in Vesiclepedia. (**c**) Top 50 abundant proteins in ectosomes include GSTP1, RAB(s) and ribosomal proteins.

Whilst an exosomal protein signature exists [3], the proteome composition of ectosomes remains uncharacterized. For this reason, a literature survey was conducted on ectosomes and molecules reported to be implicated in ectosome biogenesis or identified in ectosomes were manually curated (Supplementary Table 2). The list was later plotted as a histogram based on the fold change of the protein in exosomes compared to ectosomes or 10K. From the histograms, no enrichment could be observed for the so-called “ectosome associated proteins” except for MMP2 (Fig. 4e), a protease in the extracellular matrix. Ectosomes containing MMP2 is postulated to promote invasion when taken up by recipient cells [26]. Irrespective of the detection of MMP2 in this proteomics screen, the analysis emphasizes the need to characterize and profile the ectosomes so as to identify bona fide markers. To validate some of the findings obtained by proteomics analysis, we subjected exosomes, ectosomes, 10K and WCL fractions to Western blotting. Consistent with the proteomics analysis, exosomes exclusively contained CD81 (Fig. 5a). Even though CD63 was enriched in exosomes, it was also identified in ectosomes. MMP2, an ectosomal associated protein, was exclusively identified in ectosomes and 10K and was not be detected in exosomes.

Several other known exosomal proteins (compared to Vesiclepedia [7]) were also identified in our proteomic analysis (Fig. 5b). For instance, ADAM10 was uniquely identified in exosomes (30-fold and 10-fold enriched in exosomes compared to ectosomes and 10K, respectively). ADAM10 is involved in the cleavage of the adhesion molecule L1 at the cell surface and in EVs, suggesting a vesicle-based protease activity [27]. In addition, Plexin B, a known exosomal protein [28], was 27-fold enriched in exosomes compared to ectosomes. Similarly, GSTP1, RAB2A, RAB8B, TKT, VIM and many ribosomal proteins were in the top 50 most abundant proteins identified in ectosomes and also identified in Vesiclepedia (Fig. 5c).

### Exosomes are enriched with receptors and kinases

**Figure 6:**
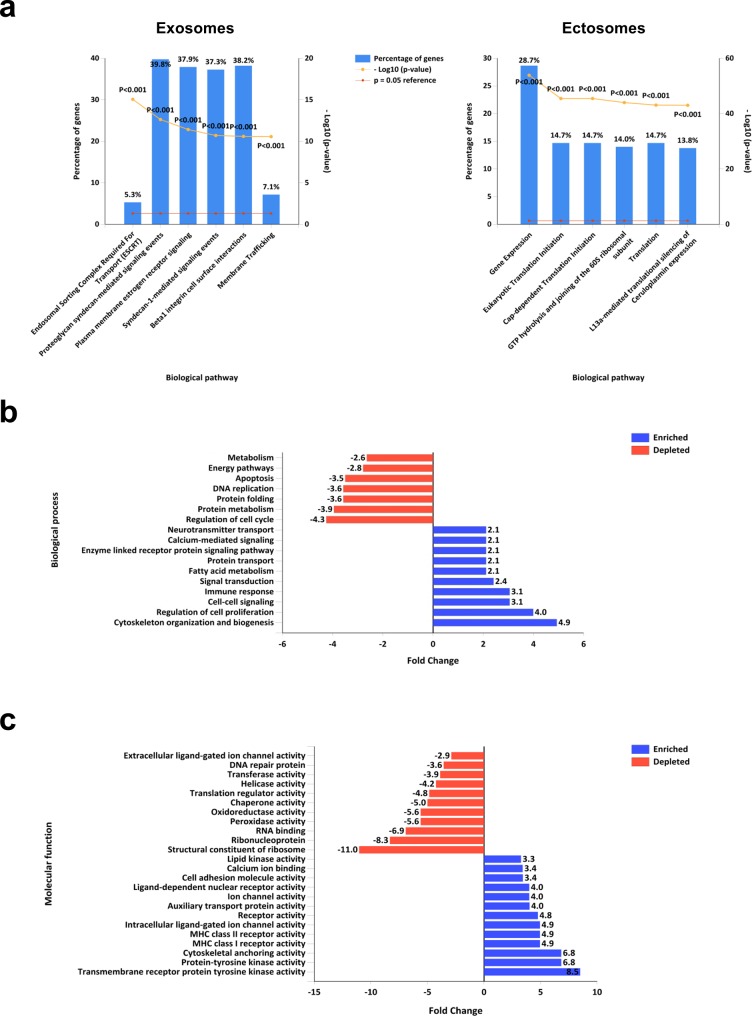
Functional enrichment analysis of exosomes and ectosomes using FunRich (**a**) Biological pathways enriched in proteins that are more than 2-fold abundant in exosomes compared to ectosomes are displayed. Proteins implicated in ESCRT, syndecan signaling and membrane trafficking are enriched in exosomes. When proteins that are more than 2-fold abundant in ectosomes compared to exosomes were analysed using FunRich, proteins implicated in gene expression and translation were enriched in ectosomes. (**b**) Gene Ontology-based biological processes that are enriched and depleted in proteins differentially abundant in exosomes compared to ectosomes are displayed. (**c**) Gene Ontology-based molecular functions that are enriched and depleted in proteins differentially abundant in ectosomes compared to exosome are displayed.

Proteins highly abundant (>2-fold) in exosomes (693) and ectosomes (770) were subjected to functional enrichment analysis using FunRich (http://www.funrich.org) software. Fold enrichment was calculated by comparing exosomal proteins against ectosomal proteins as background. In biological pathways, exosomes are enriched with proteins implicated in ESCRT, syndecan mediated signaling events, plasma membrane based signaling events, beta1 integrin cell surface interactions and membrane trafficking (Fig. 6a). In contrast, ectosomes are enriched with proteins associated with gene expression and translation (Fig. 6a). In the context of biological processes, exosomes are enriched with proteins regulating cytoskeleton organization and biogenesis, cell proliferation, cell-cell signaling, signal transduction, immune response, protein transport, fatty acid metabolism and calcium mediated signaling (Fig. 6b). On the contrary, exosomes are depleted with proteins regulating cell cycle, metabolism, protein folding, apoptosis, DNA replication and energy pathways (Fig. 6b). Consistent with these observations, molecular function-based analysis revealed the enrichment of receptors and kinases in exosomes (Fig. 6c) while depleted in ribosomal activity. The analysis also highlighted the depletion of enzymes including peroxidases, oxidoreductases, helicases and transferases in exosomes (Fig. 6c). These results allude that exosomes are enriched with signaling proteins while ectosomes are enriched with enzymes. Based on the protein cargo, it can be speculated that these two EV subtypes may have different roles in physiological and pathological conditions presumably with minimal functional redundancy.

### Ectosomes are enriched with centrosomal, ribosomal and mitochondrial proteins

As a comparison of ectosomes from two or more cell lines would provide an ectosomal protein signature, we isolated ectosomes by OptiPrep^TM^ density gradient centrifugation from SK-N-BE2 neuroblastoma cells. Since 10K pellet can serve as a crude ectosome sample, proteins identified in OptiPrep^TM^ density gradient centrifugation may also be identified in 10K pellet. While OptiPrep^TM^ density gradient centrifugation is time consuming and requires huge amounts of starting material, 10K pellet may serve as a robust sample for validating the presence of ectosomal proteins. Hence, 10K pellet from SH-SY5Y and colorectal cancer cell line LIM1215 were isolated. The ectosomes and 10K pellet samples were subjected to label-free quantitative proteomics analysis. Proteins commonly identified in ectosomes and 10K pellet but not identified in exosomes were shortlisted and analysed (Supplementary Table 3). Among these, RACGAP1, MUC19, UBR4, KRT18, KIF14, KIF4A, VIM, RPS9, RPS18, MMP2 and STAT1 were highly enriched in ectosomes and 10K samples from 3 different cell lines (SH-SY5Y, SK-N-BE2 and LIM1215). Proteins more than 2-fold highly abundant in both SH-SY5Y and SK-N-BE2 ectosomes compared to exosomes were subjected to functional enrichment analysis using FunRich tool. The analysis highlighted that ectosomes are enriched with proteins localised to centrosome, ribosome, nucleolus, cytoplasm and mitochondria (Supplementary Fig. 2a). Molecular function-based Gene Ontology analysis revealed the enrichment of proteins implicated in translation and structural constituent of ribosomes while rest of the categories (even though significant) are represented by low number of proteins (Supplementary Fig. 2b).

### Proteogenomic analysis reveals the oncogenic landscape of exosomes and ectosomes

**Figure 7:**
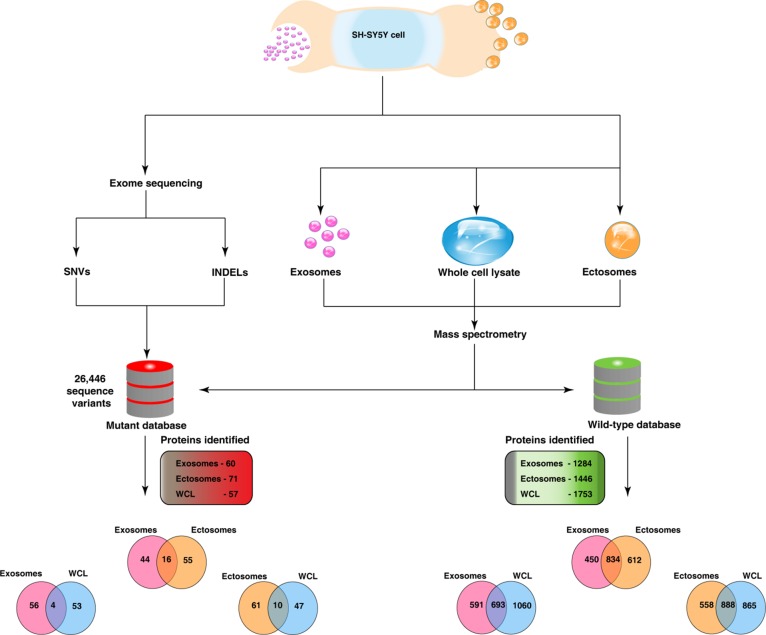
Integrated genomics and proteomics workflow This figure depicts the overview of the proteogenomics analysis involving genomic data from exome sequencing and proteomic data derived from mass-spectrometry. Exosomes and ectosomes were isolated from SH-SY5Y cells and were subjected to label-free quantitative proteomics analysis. Exome sequencing was carried out in SH-SY5Y cells and the SNVs and INDELs detected were used to create a customised mutant protein database. Proteomics analysis was also carried on SH-SY5Y WCL samples. MS/MS spectra from exosomes, ectosomes and WCL samples were searched against the wild-type and mutant database. Two-way Venn diagrams were plotted to depict the overlap of wild-type and mutant proteins identified in two respective samples. A total of 60 and 71 mutant proteins were identified in exosomes and ectosomes, respectively. Venn diagram depicting the wild-type proteins represents all identified proteins and relative abundance is not taken into account.

**Figure 8:**
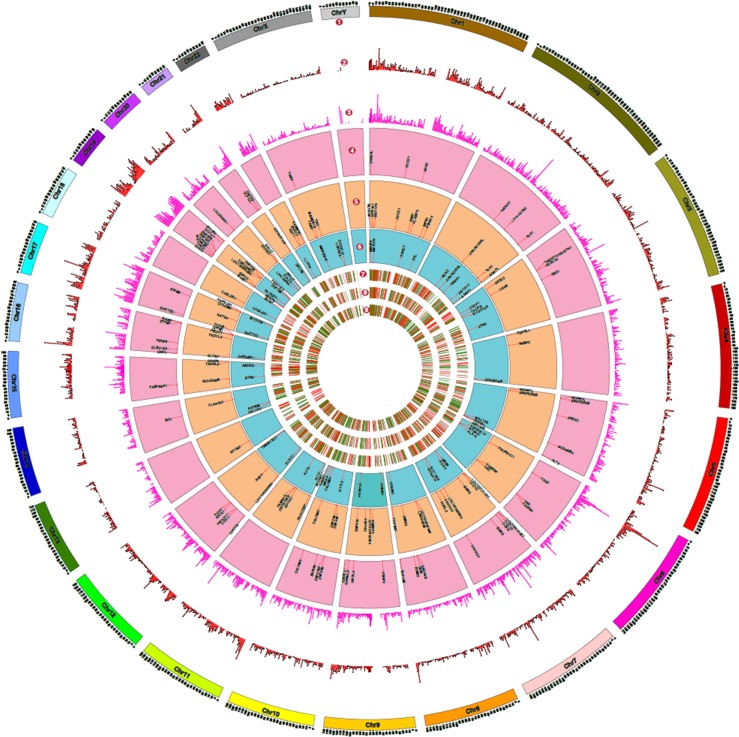
Proteogenomics landscape of exosomes, ectosomes and WCL of SH-SY5Y cells This figure depicts the overview of the genomic data derived from exome sequencing and proteomic data obtained by mass spectrometry. (1) The chromosomes represent the human ideogram. (2) Represents histogram density (every 1 Mb size) of single nucleotide variations (SNVs). (3) Represents histogram density (every 1 Mb size) of insertions and deletions (INDELs) identified using exome sequencing data. (4-6) Represents mutant proteins identified by mass spectrometry in exosomes, ectosomes and WCL, respectively, when searched against mutant databases containing the SNVs and INDELS. (7) and (8) represents heat map of differentially expressed proteins in exosomes and ectosomes in comparison to WCL, whereas (9) represents heat map of differentially expressed proteins in exosomes compared to ectosomes.

Recent studies have highlighted the secretion of oncoproteins including mutant proteins via EVs including exosomes [29, 30]. However, a prior knowledge of the mutant protein is a prerequisite in all of the published studies. A global approach to systematically identify mutant proteins secreted through EVs will aid in elucidating the functional roles of EVs. In order to identify the mutant proteins that are secreted by a cell via EVs, we adopted a global proteogenomics approach [31, 32]. Using exome sequencing, a total of 17,269 INDELs (Supplementary Table 4) and 46,842 SNVs (Supplementary Table 5) were identified in the SH-SY5Y neuroblastoma cells. Many genomic features (exonic, intronic, UTR3, UTR5, intergenic) both at the levels of SNVs and INDELs were identified (Supplementary Table 6). A customized mutant protein database with 26,446 sequence variations (non-synonmous SNVs and INDELs) was constructed using the exome sequencing data (human RefSeq protein sequences as reference) as described previously [33]. The MS/MS spectra files obtained from the exosomes, ectosomes and WCL samples were searched against the customized mutant protein database using X!Tandem search engine. Using this integrated genomics and proteomics approach (Fig. 7), a total of 60, 71 and 57 mutant proteins (Supplementary Table 7 and 8) were identified in exosomes, ectosomes and WCL, respectively. The results obtained from exome sequencing data, mutant proteins and abundance of all identified proteins are depicted in the circos plot (Fig. 8).

Furthermore, genes that are catalogued in COSMIC database for neuroblastoma were downloaded and compared with the mutant proteins secreted via exosomes and ectosomes. Among the neuroblastoma genes, RAD54B, BBS9 and UNKL were detected in exosomes while BBS9, IGFN1 and PKD1L3 were identified in ectosomes. Next, the identified mutant proteins were compared to the study by Pugh *et al.* [34], where the genetic landscape of high-risk neuroblastoma was profiled by combined whole-exome, genome and transcriptomic sequencing of 240 neuroblastoma patient samples. SIX1, a transcription factor, is mutated in SH-SY5Y neuroblastoma cells and also detected in the neuroblastoma genomic landscape study. Interestingly, the mutant protein is secreted via exosomes exclusively by SH-SY5Y cells. SIX1 is implicated in inducing proliferation, epithelial-to-mesenchymal transition, invasion and resistance to paclitaxel [35-37]. In addition, it is also proposed as a potential biomarker for gastric and pancreatic adenocarcinoma [38, 39]. The secretion of an oncogenic molecule such as SIX1 highlights the role of exosomes in cancer progression and elucidates their utility as a reservoir of disease biomarkers. Apart from SIX1, exosomes also exclusively contained mutant FLT4, FRS3 and GEM. FLT4 is a VEGF receptor that is implicated in angiogenesis [40] while FRS3 is known to regulate prostate cancer progression [41]. Likewise, GEM is a small GTP-binding protein that regulates the morphologically differentiation of neuroblastoma cells and Rho-Rho kinase pathway [42, 43]. In addition, exosomes exclusively contained mutant ICAM2, BANP and KDM4B all of which are implicated in oncogenesis. Moreover, FZD6, a Wnt receptor that is associated with the poor survival of neuroblastoma patients and resistance to doxorubicin, was also exclusively secreted through the exosomes [44].

On the other hand, mutant BIRC7, GGT1, AQP5, CABLES1, PTPN14 and NR2C2 were exclusively identified in ectosomes. BIRC7 is an apoptotic inhibitor whose expression levels are correlated with poor prognosis of neuroblastoma patients [45]. Similarly, GGT1 is implicated in pancreatic cancer by genome-wide association studies [46] while AQP5 [47] and CABLES1 [48] enhance tumour progression. PTPN14 is a tyrosine phosphatase attributed in oncogenesis and is mutated in multiple cancer types [49] while NR2C2 is known to protect neuroblastoma cells from chemotherapeutic drugs such as doxorubicin and etoposide [50]. From these results, it is evident that mutant proteins are secreted via both exosomes and ectosomes. Even though 16 mutant proteins were commonly detected in exosomes and ectosomes (Fig. 7), 99 proteins were exclusively detected in either one of the EV subtypes. Most importantly, secretion of the above mentioned mutant proteins through EVs significantly increases the role of EVs in mediating oncogenicity and drug resistant properties to recipient cells.

### Exosomes protein cargo is more oncogenic than ectosomes

**Figure 9:**
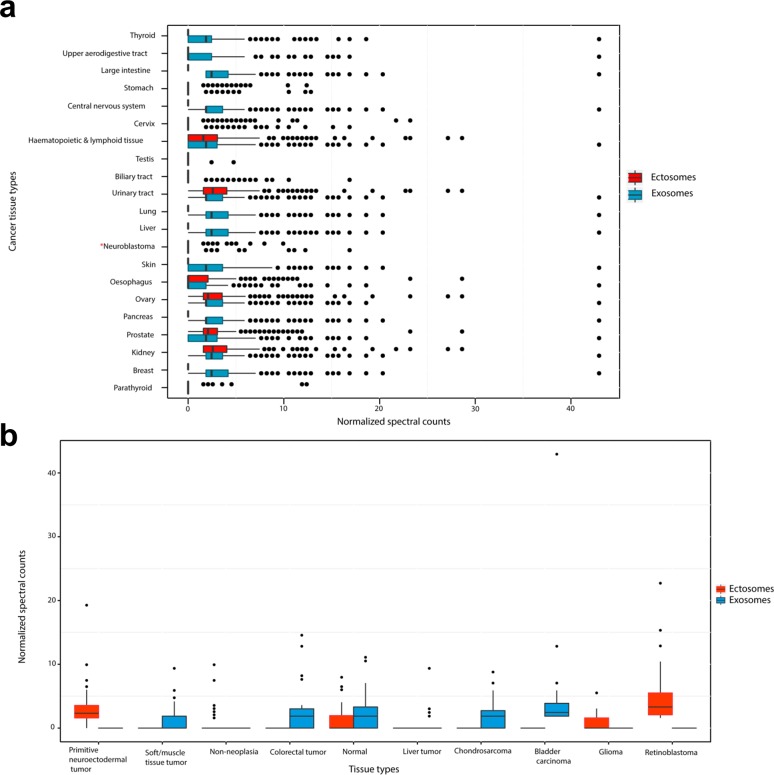
Oncogenic potential of exosomes and ectosomes (**a**) Genes significantly enriched in COSMIC database (p<0.05, chi-square test in different human cancer tissue types (except neuroblastoma which is not significantly enriched-represented by *)) were compared against proteins differentially abundant in exosomes and ectosomes. The normalized spectral counts of the enriched proteins were plotted as a box plot and grouped by the human cancer tissue types. The X-axis represents normalized spectral counts of significantly enriched (p<0.05, chi-square test) proteins whereas Y-axis represents cancer tissue types from COSMIC. Proteins identified in exosomes were implicated in many cancer types as compared to ectosomal proteins. (**b**) Genes enriched in EST database (NCBI UniGene; p<0.05, chi-square test) were compared against proteins differentially abundant in exosomes and ectosomes. The normalized spectral counts of the proteins were plotted as a box plot and grouped by the tissue types. The Y-axis represents normalized spectral counts of significantly enriched proteins whereas X-axis represents tissue types from EST database. Proteins identified in exosomes were implicated in a wide range of cancer types as compared to ectosomal proteins.

Whilst the proteogenomics analysis elucidated the secretion of mutant proteins through EVs, quantitative differences between exosomes and ectosomes pertaining to tumorigenesis could not be achieved. To investigate the oncogenic potential of exosomes and ectosomes, proteins exclusively identified in exosomes or ectosomes were compared against COSMIC and EST data set and enrichment analysis performed. When compared with COSMIC, proteins identified in exosomes were significantly enriched in multiple cancer types including thyroid, upper aerodigestive tract, large intestine, stomach, central nervous system, cervix, haematopoietic and lymphatic tissue, testis, biliary and urinary tracts, liver, lung, skin, oesophagus, ovary, pancreas, prostate, kidney, breast and parathyroid (Fig. 9a). However, proteins identified in ectosomes were enriched in a subset of these cancer types such as stomach, kidney, cervix, oesophagus, parathyroid, urinary tract, prostate, haematopoietic and lymphoid tissue and ovary. Further to this, when the protein abundance of the enriched genes was depicted as a box plot (Fig. 9a), exosomal proteins were highly abundant in most of the cancer types compared to ectosomal proteins. In agreement with this, when EST data sets were compared, exosomal proteins were more abundant in a wide range of cancer types (Fig. 9b). In contrast, ectosomal proteins were more abundant primarily in retinoblastoma and primitive neuroectodermal tumor. Furthermore, 28% (127) of the proteins uniquely identified in exosomes were significantly enriched (chi-square test, p=0.0002) in the genes that are known to be mutated in the high-risk neuroblastoma genomic landscape study [34]. On the contrary, the proteins uniquely identified in ectosomes were not significantly enriched (p=0.08) when compared to the neuroblastoma genomic landscape study. Consistent with these observations, exosomal proteins were also significantly enriched (p=0.02) in Cancer Gene Census data set (COSMIC) while ectosomal proteins were not. Overall, these results suggest that exosomal cargo contain more oncoproteins than ectosomes.

### Exosomes induce more proliferation and migration of target cells than ectosomes

**Figure 10:**
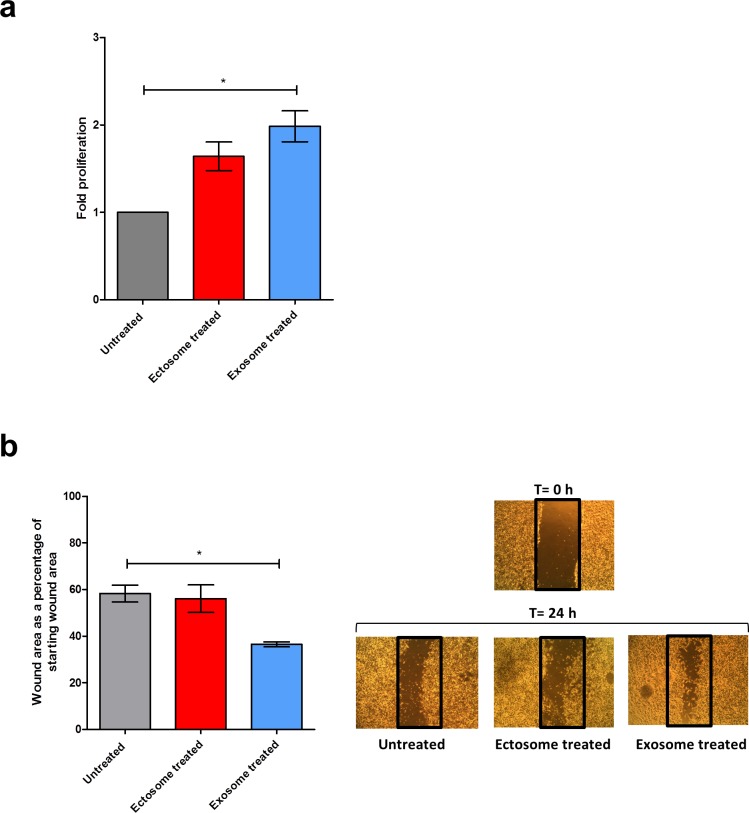
Cell proliferation and migration potential of exosomes and ectosomes (**a**) MTS cell proliferation assay was performed with SK-N-BE2 neuroblastoma cells treated with exosomes and ectosomes derived from SH-SY5Y cells for 24 h. As a control, untreated SK-N-BE2 cells were grown. Exosomes induced a 2-fold proliferation of SK-N-BE2 cells. On the contrary, ectosomes did not induce any significant proliferation of SK-N-BE2 cells. Error bars represent standard error of the mean, n=3, * denotes significance (p<0.05). (**b**) Wound healing assay of neuroblastoma cell line SK-N-BE2 is displayed. Wound was created post reaching 100% confluence, and cells were treated with either exosomes or ectosomes for 24 h. Migration was assessed at 24 h after wounding. Images were taken under the 4x objective of the light microscope. Quantification of wound closure showed that exosomes induced more migration compared to ectosomes. Error bars represent standard error of the mean, n=3, * denotes significance (p<0.05). Student's t-test was used to evaluate statistically significant differences between the values.

Whilst the bioinformatics analysis highlighted the oncogenic potential of exosomes, biochemical experiments would further validate these observations. To assess the oncogenic potential, MTS assay was performed to evaluate the capacity of exosomes and ectosomes to stimulate proliferation in recipient cells. SK-N-BE2 neuroblastoma cells were treated with and without exosomes or ectosomes isolated from SH-SY5Y cells. A significant difference (2-fold) in cell proliferation and metabolic flux was observed when SK-N-BE2 cells were treated with exosomes compared to untreated cells (Fig. 10a). Even though treatment of ectosomes induced cell proliferation in SK-N-BE2 cells, the events were not statistically significant. Further to cell proliferation, the role of exosomes and ectosomes in cell migration was studied. Wound healing assay was performed by making a uniform scratch on a monolayer of SK-N-BE2 cells at 100% confluence. The scratches were then observed at different time points for closure of the wound gap (Fig. 10b). Interestingly, the wound closure rate of SK-N-BE2 cells was faster upon exosome treatment (p<0.05). However, ectosomes treatment did not result in a significant change in cell migration compared to untreated cells. Consistent with the bioinformatics analysis, these results reassert that exosomes can induce more proliferation and migration of target cells compared to ectosomes.

## DISCUSSION

Whilst EVs have attracted significant interests from the scientific community, few challenges need to be resolved [11]. One of them attribute to the need of reliable markers that can distinguish between exosomes and ectosomes [12]. Currently standardized EV isolation and purification methods fail to separate exosomes from ectosomes and vice versa [8]. In this study, we isolated exosomes from SH-SY5Y neuroblastoma cells by OptiPrep^TM^ density gradient centrifugation. Ectosomes were isolated from higher density fractions of OptiPrep^TM^ density gradient centrifugation as well as by the simple 10,000 *g* centrifugation (crude). Exosomes floated at 1.10 g/mL while ectosomes float between 1.14-1.20 g/mL. As expected, microscopic analysis confirmed the presence of smaller vesicles (30-100 nm in diameter) in exosome fractions and larger vesicles (>200 nm in diameter) in ectosome samples. Even though the isolated fractions were enriched with vesicles of expected size (small or large), the analysis also highlighted that exosomes contained a minor population of large vesicles (>150 nm in diameter) and vice versa. Hence, we emphasize that the isolated EV subtypes were enriched for either exosomes or ectosomes and are not 100% pure populations of any one EV subtype. Label-free quantitative proteomics analysis on the EV subtypes revealed some of the markers that could discriminate between exosomes and ectosomes. As expected, exosomes were enriched with ESCRT components, tetraspanins, annexins, flotillins and integrins. Most importantly, exosomes exclusively contained VPS24, VPS32 and VPS36 of the ESCRT components, CD81, TSPAN9 and TSPAN14 of tetraspanins, ANXA7, Syntenin (SDCBP) and ITGA3. On the contrary, proteins exclusively present in ectosomes include RACGAP1, PDIA3, SPTBN2, MUC19, UBR4, KRT18, KIF14, KIF4A, VIM, RPS9, RPS18 and MMP2. While some of these proteins may be specific to the cell type of origin, a subset of these proteins can be exploited as markers of ectosomes. Based on proteomics and Western blot results, CD81 and MMP2 can be used as exosomal and ectosomal markers, respectively.

Whilst the proteins identified in exosomes elucidated some of the key proteins implicated in exosome biogenesis and cargo sorting, ectosomal cargo did not provide compelling data in the context of ectosome biogenesis. In spite of not being packaged into ectosomes, some proteins may still regulate the biogenesis. For instance, ARF6 is thought to regulate ectosome biogenesis [10, 51] and could not be detected in this proteomics screen. One of the interesting observations in this study relate to the exclusive identification of a subset of RABs in ectosomes and 10K. To further understand the biogenesis of ectosomes, more focussed studies need to be performed to underpin the role of cellular proteins including RABs. From the functional enrichment analysis, exosomes were enriched with more membrane proteins compared to ectosomes while ectosomes contained more intracellular proteins. It is tempting to speculate that exosomes could potentially orchestrate cell communication and signal transduction pathways better than ectosomes. However, as exosomes are comparatively smaller than ectosomes, the surface area of exosomes is much smaller. Hence, for equal amounts of protein, the chance of identifying membrane proteins is higher in exosomes compared to ectosomes. Studies based on equal number of vesicles may provide valuable information on the proteomic cargo.

One of the novel features of this study relates to the global proteogenomics analysis of exosomes and ectosomes. For the first time, the analysis allowed for the identification of mutant proteins that can be secreted via exosomes and ectosomes. Protein cargo sorting into EVs, especially exosomes, is often debated for the selectivity or randomness of the process. In this study, compared to the WCL, low abundant proteins are highly enriched in exosomes or ectosomes. This suggests that molecular machinery may exist within the cell that can selectively package oncoproteins into exosomes/ectosomes. Identified mutant proteins that are exclusively secreted via exosomes include SIX1, FZD6, FLT4, FRS3, GEM, ICAM2, BANP and KDM4B all of which are implicated in oncogenesis and/or drug resistance. Similarly, mutant BIRC7, GGT1, AQP5, CABLES1, PTPN14 and NR2C2 were exclusively identified in ectosomes. The transfer of these oncoproteins and mutant proteins via exosomes/ectosomes highlights the pivotal role of EVs in various disease conditions including cancer. As EVs are secreted/released into the extracellular microenvironment and can be assessed in bodily fluids [52], they are considered as reservoirs of potential biomarkers [3]. Current protein/RNA based biomarker studies often assay for wild type forms [53]. Assaying for mutant protein/RNA as disease biomarkers provides the required specificity for a biomarker test as mutant protein/RNA are encoded by diseased cells. Thus, the secretion of mutant proteins via EVs provides unparalleled opportunity to assay for them non-invasively in patient bodily fluids.

Integrated bioinformatics and experimental approach revealed that exosomes are enriched in oncogenic cargo and can induce cell proliferation and migration more than ectosomes. Considering all the results, it can be speculated that the two EV subtypes, exosomes and ectosomes, may have distinct functionalities. As exosomes and ectosomes have some proteins in common, a functional redundancy in certain cases cannot be excluded. We strongly believe that the integrative proteogenomics approach utilized in this study will uncover many novel insights and allow choosing disease biomarker candidates when applied on additional EV studies.

## MATERIALS AND METHODS

### Cell culture

Human neuroblastoma cell lines SH-SY5Y and SK-N-BE2 were gifted by Dr. Julie Atkins (Department of Biochemistry, La Trobe University, Australia) and Dr. Loretta Lau (Sydney Medical School, University of Sydney, Australia), respectively. LIM1215 colorectal cancer cell lines were obtained from Ludwig Institute of Cancer Research, Australia. Neuroblastoma cells (SH-SY5Y and SK-N-BE2) and colorectal cancer cells (LIM1215) were cultured in DMEM and RPMI medium, respectively, in the presence of 10% FCS (GIBCO, Life Technologies) and 100 Units/mL of penicillin-streptomycin (GIBCO, Life Technologies). The cells were cultured in 5% CO_2_ atmosphere at 37ºC.

### Preparation of conditioned media (CM)

For preparing CM, cells were seeded in 150 mm diameter culture dishes in the presence of 25 mL of culture medium. After cell density reached 70-80% confluence, the cells were washed thrice with serum free media. Cells were then cultured in 15 mL of DMEM or RPMI supplemented with 0.6% insulin transferrin selenium (ITS) and 100 Unit/mL of penicillin-streptomycin for 24 h. The CM was collected, centrifuged at 500 *g* for 10 min to remove floating cells followed by another centrifugation step at 2,000 *g* for 20 min.

### Differential centrifugation coupled ultracentrifugation

The supernatant collected after the 2,000 g spin was then subjected to ultracentrifugation at 100,000 g (SW-40 rotor, Beckman) for 1 h at 4°C to pellet the EVs. The crude extract of EVs were then stored in −80°C for further use. For crude ectosomes, the CM was subjected to 10,000 g for 30 min and the pellet obtained was washed with PBS and stored in −80°C.

### OptiPrep™ density gradient centrifugation

Iodixanol based separation solution was used to isolate pure population of exosomes and ectosomes from the crude extract. The self-generated fractions were composed of 40%, 20%, 10% and 5% (w/v) dilutions of iodixanol (Axis-Shield PoC) buffered with 0.25 M sucrose and 10 mM Tris (pH 7.5). SH-SY5Y crude pellets were resuspended in the OptiPrep™ solution and overlaid onto the top layer. A control tube consisting 3 mL of each 40%, 20%, 10% and 5% solutions were also prepared. The tubes were subjected to centrifugation at 100,000 g (SW-28 rotor, Beckman) for 18 h at 4°C. Pellets were then washed with 1 mL of PBS and resuspended in 200 μL before being stored at −80°C.

### Apoptosis assay

At the time of CM collection (containing 0.6% ITS) for EV isolation, the cells were also harvested. Additional plates of SH-SY5Y neuroblastoma cells were also included as controls for detection of levels of cell death. The CM and the harvested cells were subjected to centrifugation at 500 *g* for 5 mins. Supernatant was removed and the pellet, comprised of live and dead cells, was mixed and resuspended with 1 mL 0.4% Trypan Blue (Santa Cruz). The dead and live cells were counted using Neubauer haemocytometer (La fontaine) and cell viability was determined. The cells were grown with or without FCS and ITS and treated with 1 μM doxorubicin (Hospira Inc.) for 24 and 48 h. Cells were then harvested and lysates were subjected to Western blot analysis.

### Western blotting and antibodies

SDS-PAGE was used to separate equal amounts of protein (10 or 30 μg quantified by Sypro^®^ Ruby stain) from EVs and WCL samples. Gels were transferred to nitrocellulose membrane using an iBlot™ gel transfer stack system (Life Technologies). Membranes were then blocked with skim milk and probed overnight with the primary antibodies against TSG101 (BD Transduction Laboratories), Alix (Cell Signaling), GM130 (BD Transduction Laboratories), Caspase-3 (Cell Signaling) and Poly (ADP-ribose) polymerase-1/PARP-1 (Santa Cruz Biotech). Fluorescent conjugated rabbit and mouse secondary antibodies were used and the protein bands were visualized using ODYSSEY CLx (LI-COR^®^).

### Transmission electron microscopy

Samples (0.2 μg/μL) were examined in a JEM-2010 transmission electron microscope (JEOL, 80 kV) or Tecnai TF30 transmission electron microscope (FEI, 300 kV). Preparations were fixed to 400 mesh carbon-layered copper grids for up to 2 min. Surplus material was drained by blotting followed by negatively staining of samples with 10 μL of uranyl acetate solution (2% w/v; Electron Microscopy Services).

### Atomic force microscopy

Samples (1-4 μL) were deposited onto freshly cleaved mica surface and incubated for 15 min. The samples were consecutively rinsed with 50 μL drops of water for up to 5 times before drying under a stream of argon gas. Imaging was performed with an Ntegra AFM platform (NT-MDT, Zelenograd, Russia). For imaging NSC15 (MikroMasch) and NSG10 (NT-MDT) probes were used in intermittent contact mode with typical resonance frequencies within the range of 200-400 kHz. For image processing, Gwyddion freeware (www.gwyddion.net) was used. Standard image processing steps included plane background subtraction, offset flattening, as well as polynomial background subtraction and Gaussian filtering (2 pixels) as required.

### In gel digestion

Equal amount (20 μg) of exosomes, ectosomes and WCL fractions were electrophoretically separated using SDS-PAGE and proteins were visualized by staining with Coomassie Brilliant Blue stain. Gel lanes were cut into 20 × 2 mm bands using a scalpel blade and proteins were reduced, alkylated and trypsinised as described previously [54]. Briefly, the gel bands were subjected to reduction by 10 mM DTT (Bio-Rad), alkylation by 55 mM iodoacetamide (Sigma) and tryptic digestion overnight with 150 ng of trypsin (Promega). Subsequently, the tryptic peptides were further extracted using 0.1% trifluoroacetic acid in acetonitrile (50% w/v).

### LC-MS/MS

Extracted tryptic peptides from each gel band were concentrated to ~10 μL by centrifugal lyophilisation and analysed by LC-MS/MS using LTQ Orbitrap Velos mass spectrometer (Thermo Scientific) fitted with nanoflow reversed-phase-HPLC (Model 1200, Agilent). The nano-LC system was equipped with an Acclaim Pepmap nano-trap column (Dionex – C18, 100 Å, 75 μm × 2 cm) and an Acclaim Pepmap RSLC analytical column (Dionex – C18, 100 Å, 75 μm × 15 cm). Typically for each LC-MS/MS experiment, 1 μL of the peptide mix was loaded onto the enrichment (trap) column at an isocratic flow of 3 μL/min of 3% CH3CN containing 0.1% formic acid for 4 min before the enrichment column is switched in-line with the analytical column. The eluents used for the LC were 0.1% v/v formic acid (solvent A) and 100% CH3CN/0.1% formic acid v/v. The gradient used was 3% B to 8% B for 1 min, 8% B to 35% B in 30 min, 35% B to 85% B in 5 min and maintained at 85% B for the final 5 min. All spectra were acquired in positive mode with full scan MS spectra scanning from m/z 300–2000 in the FT mode at 30,000 resolution after accumulating to a target value of 1.00e6 with maximum accumulation of 500 ms. The 20 most intense peptide ions with charge states ≥2 were isolated at a target value of 1000 and fragmented by low energy CID with normalized collision energy of 30 and activation Q of 0.25. Dynamic exclusion settings of 2 repeat counts over 30 s and exclusion duration of 70 s.

### Database searching and protein identification

Peak lists were generated using extract-msn as part of Bioworks 3.3.1 (Thermo Scientific) using the following parameters: minimum mass 300; maximum mass 5,000; grouping tolerance 0.01 Da; intermediate scans 200; minimum group count 1; 10 peaks minimum and total ion current of 100. Peak lists for each LC-MS/MS run were merged into a single mascot generic format. Automatic charge state recognition was used because of the high resolution survey scan (30,000). LC-MS/MS spectra were searched against the NCBI RefSeq human protein database [55] in a target decoy fashion using X!Tandem Sledgehammer (2013.09.01.1). Search parameters used were: fixed modification (carboamidomethylation of cysteine; +57 Da), variable modifications (oxidation of methionine; +16 Da and N-terminal acetylation; +42 Da), three missed tryptic cleavages, 20 ppm peptide mass tolerance and 0.6 Da fragment ion mass tolerance. Protein identifications with at least 2 unique peptides were shortlisted to obtain a master list with less than 0.5% false discovery rate.

### DNA extraction and exome sequencing

Around 5 × 10^6^ cells were harvested by trypsinisation and washed with PBS. The genomic DNA was extracted from the cell pellet using PureLink Genomic DNA mini kit (Invitrogen) according to the manufacturer's instructions. Briefly, lysate was subjected to Proteinase K digestion at 55°C and removal of residual RNA by RNase digestion. The lysate was mixed with ethanol and PureLink^®^ Genomic Binding Buffer that allows high DNA binding to the silica-based membrane in the PureLink^®^ Spin Column. Impurities are removed by washing with Wash Buffers 1 and 2. The genomic DNA is then eluted in low salt Elution Buffer. The purified genomic DNA was quantified at A260/A280 (Nano Drop ND-1000 spectrometer, Biolabs). The protocol yielded >4 μg of purified genomic DNA which was used to prepare exome captured sequencing library using Illumina TrueSeq Exome Enrichment kit. Sequencing of exome capture library was carried out by Australian Genome Research Facility Ltd. (AGRF), Melbourne, using Illumina HiSeq 2000. In total, ~62 Mb of genomic sequence was targeted. Sequencing of 100-bp paired-end reads was performed. SNVs and INDELs were detected using ANOVAR tool.

### Mutant database construction

All the coding non-synonymous single nucleotide variations (cSNVs) predicted by ANOVAR tool were searched against the human genome annotation release 105. The corresponding protein sequences with respective mutations were created using in-house Python scripts. In addition, INDELs predicted by ANOVAR tool were searched against the human genome annotation release 105. Up to 150 nucleotide sequences upstream and downstream from the INDEL positions were fetched and translated into six reading frames using in-house Python scripts. With the translated sequences, a mutant protein database was created. The resulting database was queried using the MS/MS spectra of exosomes, ectosomes and WCL using X!Tandem Sledgehammer (2013.09.01.1) search engine [56].

### Label-free spectral counting

The relative protein abundance between the samples was obtained by estimating the ratio of normalized spectral counts (RSc) as previously described [57].

RSc for protein A = [(*sY+c*) (*TX-sX+c*) / (*sX+c*) (*TY-sY+c*)]

Where *s* is the significant MS/MS spectra for protein A, *T* is the total number of significant MS/MS spectra in the sample, *c* is the correction factor set to 1.25, and *X* and *Y* are the exosome and ectosome samples, respectively. When RSc is less than 1, the negative inverse RSc value was used.

### Polar histogram

Polar Barchart modified from Polar Histogram package (http://www.jstatsoft.org/v40/i01/) was downloaded and installed in R v3.0.2 (http://www.R-project.org/). Top 50 proteins (based on their abundance value) identified in exosomes and ectosomes were used to fetch the number of experimental studies reported in Vesiclepedia. The mapped experimental studies along with their protein abundance value for all the top 50 proteins were plotted using Polar Barchart package implemented in R.

### Functional enrichment analysis

Proteins identified in exosomes, ectosomes and WCL were subjected to Gene Ontology (GO) and biological pathway enrichment analysis using FunRich tool (http://www.funrich.org) against human FunRich background database.

### Circos diagram

Circos (v0.67), an open source software tool to represent genomes, was downloaded and used to depict the overall exome sequencing derived genomics data of SH-SY5Y cells and mass spectrometry-derived proteomics data of exosomes, ectosomes and WCL. SNVs and INDEL density files for the Circos were generated using in-house Python scripts.

### Oncogenic profiling

Mutation data annotated in different cancer tissue types in COSMIC v70 (Catalogue of somatic mutations in cancer) was downloaded [58]. Proteins exclusively identified in exosomes and ectosomes were checked for the enrichment of oncogenic genes by comparing against the COSMIC background database. The normalized spectral counts of the significantly enriched genes (Chi-square test, p<0.05) in different human cancer types were further depicted as boxplot. In addition, data sets from EST (NCBI-UniGene) cDNA libraries from different tumor and normal tissue types were further downloaded. The expression abundance in the form of transcript per million (TPM) counts for each gene across different tissue types was calculated and ranked as described previously [59]. Using this as a background database, the highly abundant and depleted proteins identified in exosomes and ectosomes were compared to check for the enrichment of genes in different cancer types. The normalized spectral counts of those significantly enriched genes (Chi-square test, p<0.05) in different human tissue types were further depicted as boxplot.

### Cell proliferation assay

Equal numbers of SKN-BE2 cells were seeded in 96-well plates. After 24 h, cells were treated with 100 μg/mL of exosomes or ectosomes. The proliferation was detected by MTS assay at 0 h (t=0) and 24 h post treatment (t=24) time points. MTS solution (PMS reagent (Sigma Life Science^®^) in DPBS and CellTiter 96^®^ Aqueous MTS reagent powder in DPBS (Promega) at the ratio of 1:20, according to manufacturer's protocol) was added to each well. The plate was incubated for 1.5 h after the addition of MTS solution. The absorbance was measured using SpectraMaxM5 multi-mode microplate reader (Molecular Devices) at wave lengths 490 and 630 nm.

### Wound healing assay

Equal numbers of SKN-BE2 cells were seeded in 12-well plates and were allowed to reach 100% confluence. A pipette tip was used to scratch the monolayer of cells. Detached cells were removed by replacing the media. Cells were then incubated at 37°C in 5% CO_2_ with 20 μg/mL of exosomes or ectosomes. The width of the wound was monitored under the microscope at 0 and 24 h post treatment. ImageJ software was used to calculate the wound area.

### Statistical analysis

Statistical analysis were performed with R or Prism5 (GraphPad) or Microsoft Office Excel. All data shown are representative of results obtained from experiments conducted two or three times as specified in the respective sections. The significance of the results were analysed by T-tests or Chi-square tests.

## SUPPLEMENTARY MATERIALS FIGURES AND TABLES


















