# Supplementary material for: Proteogenomic analysis reveals exosomes are more oncogenic than ectosomes
Source: Oncotarget. 2015 Apr 12;6(17):15375–96. doi: 10.18632/oncotarget.3801 (PMC4558158; doi:10.18632/oncotarget.3801)
Supplement: Supplementary file 9 [file oncotarget-06-15375-s009.pdf]

Proteogenomic analysis reveals exosomes are more oncogenic than ectosomes

Supplementary Material

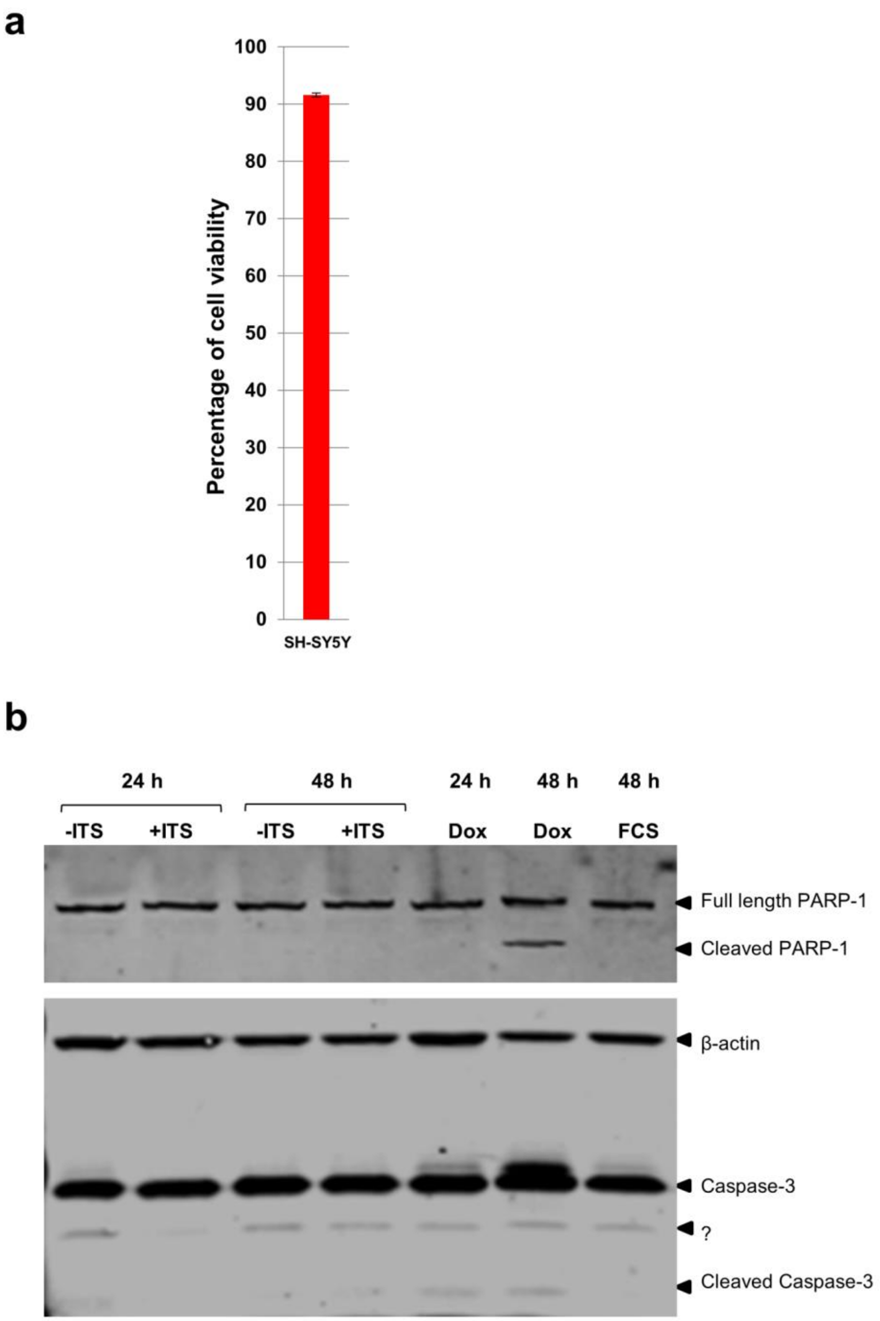

Supplementary Figure 1

Trypan blue and Western blot analysis of SH-SY5Y cells

(a) Trpan blue assay was performed to analyse the percentage of cell death in SH-SY5Y cells during exosome/ectosome collection. Close to 8.7% cell death was observed in SH-SY5Y cells. (b) SH-SY5Y cells were cultured with and without ITS for 24 and 48 h. In addition, the cells were cultured with FCS (10%) for 48 h. Cells cultured with FCS were treated with 1 µM doxorubicin for 24 and 48 h. WCL were prepared and subjected to Western blot analysis. Probing with PARP-1 and Caspase 3 highlighted the presence of cleavage and activation of these molecules in doxorubicin treated samples. An unknown nonspecific band was also observed in all WCL samples except ITS cultured cells for 24 h.

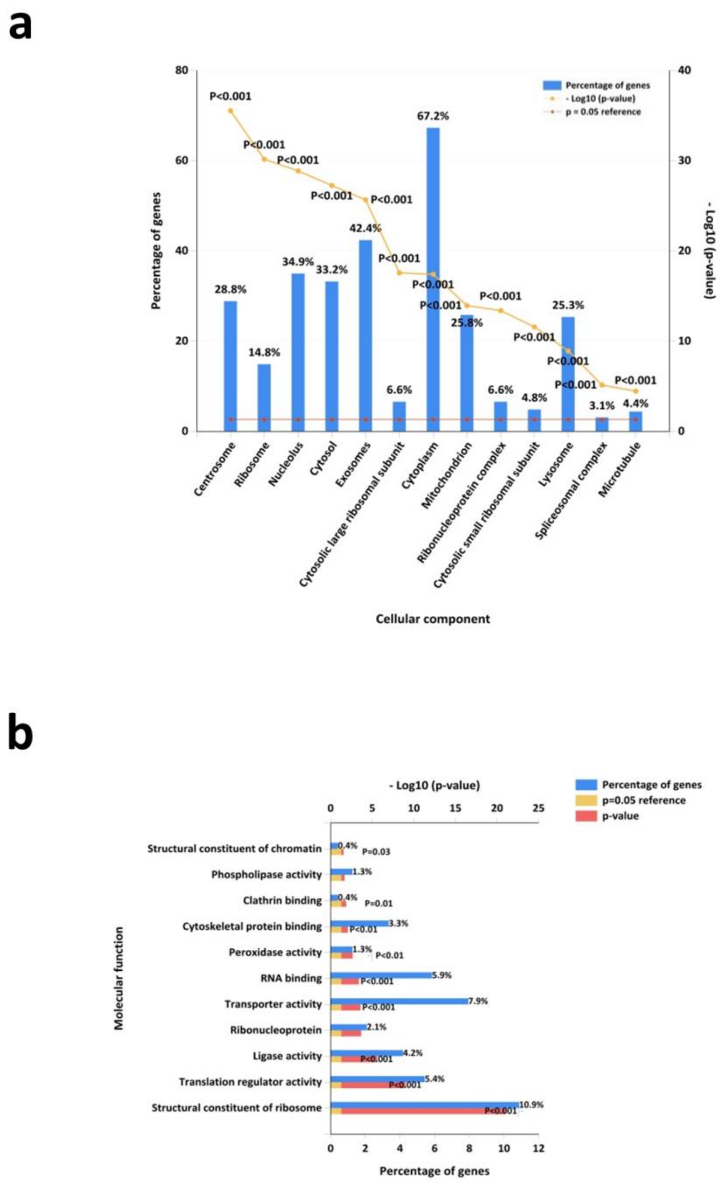

Supplementary Figure 2

Functional enrichment analysis of ectosomes using FunRich

Proteins 2-fold highly abundant in both SH-SY5Y and SK-N-BE2 ectosomes compared to exosomes were subjected to functional enrichment analysis using FunRich software. (a) Subcellular compartment-based enrichment analysis highlighted that ectosomes are enriched with proteins localised to centrosome, ribosome, nucleolus, cytoplasm and mitochondria. (b)

Molecular function-based analysis highlighted the enrichment of proteins implicated in translation and structural constituent of ribosome in ectosomes.

Supplementary Table 1

List of proteins identified in exosomes, ectosomes and WCL from SH-SY5Y neuroblastoma cells

Supplementary Table 2

A manually curated list of proteins known to be involved in ectosome biogenesis and/or identified in ectosomes

Supplementary Table 3

List of proteins uniquely identified in ectosomes/10K isolated from SH-SY5Y, SK-N-BE2 and LIM1215 cells

Supplementary Table 4

A complete list of INDELs identified by exome sequencing in SH-SY5Y neuroblastoma cells

Supplementary Table 5

A complete list of SNVs identified by exome sequencing in SH-SY5Y neuroblastoma cells

Supplementary Table 6

Statistics of SNV and INDEL features at exonic, intronic, UTR and intergenic level identified by exome sequencing in SH-SY5Y neuroblastoma cells

Supplementary Table 7

Statistics of SNVs and INDELs at exonic, intronic, UTR and intergenic level identified in mutant proteins from SH-SY5Y neuroblastoma cells

Supplementary Table 8

List of mutant proteins detected in exosomes, ectosomes and WCL from SH-SY5Y neuroblastoma cells
